# Whole-Genome Sequencing Reveals Age-Specific Changes in the Human Blood Microbiota

**DOI:** 10.3390/jpm12060939

**Published:** 2022-06-07

**Authors:** Eun-Ju Lee, Joohon Sung, Hyung-Lae Kim, Han-Na Kim

**Affiliations:** 1 Medical Research Institute, Kangbuk Samsung Hospital, School of Medicine, Sungkyunkwan University, Seoul 03181, Korea; ionantha97@gmail.com; 2 Department of Epidemiology, School of Public Health, Seoul National University, Seoul 08826, Korea; jsung@snu.ac.kr; 3 Department of Biochemistry, College of Medicine, Ewha Womans University, Seoul 07804, Korea; hyung@ewha.ac.kr; 4 Functional Genome Institute, PDXen Biosystems Inc., Daejeon 34129, Korea; 5 Department of Clinical Research Design and Evaluation, SAIHST, Sungkyunkwan University, Seoul 06355, Korea

**Keywords:** blood microbiota, whole-genome sequencing, unmapped reads, age

## Abstract

Based on several reports that indicate the presence of blood microbiota in patients with diseases, we became interested in identifying the presence of bacteria in the blood of healthy individuals. Using 37 samples from 5 families, we extracted sequences that were not mapped to the human reference genome and mapped them to the bacterial reference genome for characterization. Proteobacteria account for more than 95% of the blood microbiota. The results of clustering by means of principal component analysis showed similar patterns for each age group. We observed that the class Gammaproteobacteria was significantly higher in the elderly group (over 60 years old), whereas the arcsine square root-transformed relative abundance of the classes Alphaproteobacteria, Deltaproteobacteria, and Clostridia was significantly lower (*p* < 0.05). In addition, the diversity among the groups showed a significant difference (*p* < 0.05) in the elderly group. This result provides meaningful evidence of a consistent phenomenon that chronic diseases associated with aging are accompanied by metabolic endotoxemia and chronic inflammation.

## 1. Introduction

The microbiome, which has co-adapted with humans for millions of years, represents a complex mixture of eukaryotes, bacteria, archaea, and viruses. This microbiome significantly contributes to human physiology, by supplying nutrients and protecting against pathogens [1,2]. The human microbiome varies dynamically according to various characteristics, such as individual, family, race, gender, age, eating habits, regional lifestyle, and genetics [3,4,5]. Family-based studies offer the advantage of controlling some factors that affect the microbial community structure, thereby allowing for the study of factors influencing the microbes and abundance.

Metagenomic approaches for studying microbial genomes are being used to determine the potential roles of the gut microbiome, skin, and blood in chronic inflammatory diseases [6,7,8]. According to the inflammatory theory, inflammation underlies many chronic diseases, which means that lipopolysaccharides (LPS) from inflammatory cytokines and bacteria are present in the blood [9]. This gives rise to the probability that bacteria act as inflammatory sources and might be present in the blood even in a healthy state. Evidence of a dormant blood microbiota comes from its direct assessment using culture-independent methods [10], including the detection of blood (or tissue) microbial macromolecules such as the 16S ribosomal RNA (rRNA) gene [11] and direct visualization of cells using ultramicroscopic methods [10]. Since human blood has traditionally been thought to be a completely sterile environment composed only of blood cells, platelets, and plasma, the presence of microbes in the blood has consistently been interpreted as an indication of infection [12]. Although it is a controversial concept, there is increasing evidence for the existence of a healthy human blood microbiota [12]. The relationship between the blood microbiota and liver fibrosis has been reported in patients with severe obesity [8]. A predictive role of the blood microbiota in cardiometabolic diseases has been observed in the general population [13,14]. In line with these results, our previous study also demonstrated that lean individuals had different characteristics of the blood microbiota in the condition of non-alcoholic fatty liver disease [15].

Recently, the application of high-throughput technology to investigate complex microbial communities has yielded significant progress in the characterization of the human microbiome and its role in health and disease. Metagenomics using next-generation sequencing (NGS) is a powerful approach for identifying microbial communities because it is difficult to use culture methods to isolate more than a small percentage of micro-organisms in healthy individuals [16,17]. Recent research has focused on the development of bioinformatics tools for detecting the presence of pathogens from human sequence data, by computationally subtracting known human sequences [18,19]. The alignment of reads to a reference genome is a common approach in NGS. Unmapped reads are typically discarded from whole-genome sequencing (WGS) analysis, but potentially biologically relevant information and insights can be obtained from them [20,21]. With the wide applicability of NGS methods, there is a growing interest in the source of unmapped reads [22].

Although evidence indicating the presence of a microbial component in the blood of healthy human individuals is steadily accumulating [23,24,25], the influence of age on healthy human blood microbiota composition remains ambiguous. Aging affects both the host and microbiome physiologically, and host-microbiome interactions may affect aging [26]. Most contemporary research has focused on age-related microbiomes in the human gut [27,28]. The aim of this study was to demonstrate the presence of a blood microbiota in healthy individuals and to identify bacteria at the phylum and class levels using NGS data with 30×~100× depths produced for human genome research purposes. In the present study, we report the basic characteristics and existence of the blood microbiota through bacterial mapping of WGS unmapped reads from five families, using high-depth sequencing data. Furthermore, we investigated the changes in the blood microbiota composition of healthy subjects, according to age group.

## 2. Materials and Methods

### 2.1. Subjects

Blood samples were collected from 5 families enrolled at the Korean Family Cohort, between 2006 and 2012. The 37 subjects in this study included 18 men and 19 women, aged between 18 and 80 years old. This cohort consisted of 3-generation families, and the family members had been undergoing annual health check-ups since 2006. For this study, we selected patients who did not have infectious diseases or fever, in addition to no history of immune disease, cancer, or severe disease, in the period between 2006 and 2013. However, out of the 10 elderly people who were grandparents in each family, 1 had both diabetes and hypertension, while another had diabetes. The average body mass index (BMI) value of the 37 subjects was 23.6, and 3 persons were over 30, but they were healthy, without metabolic disease and clinical symptoms. Therefore, the experiment was conducted on individuals who did not suffer from infectious diseases or diseases with an infection-related mechanism. All participants provided written informed consent using protocols approved by the IRB of Ewha Womans University (EUMC201608013) and Kangbuk Samsung Hospital (KBSMC201508035002).

### 2.2. WGS and Materials

Genomic DNA was extracted from whole-blood samples using the QIAamp DNA Blood Mini Kit (QIAGEN, Hilden, Germany), according to the manufacturer’s protocols, within 24 h of the blood samples being collected in Vacutainer^®^ tubes (BD Biosciences, Mississauga, ON, Canada) containing ethylenediaminetetraacetic acid. The extracted genomic DNA was immediately stored in a –80 °C freezer in the laboratory, until use. Genomic DNA from each sample was subjected to WGS using an Illumina TruSeq™ DNA-seq DNA sample preparation kit (Illumina, San Diego, CA, USA) and the Illumina HiSeq X Ten platform (Illumina), according to the manufacturer’s instructions in 2014. The raw.bcl files were then converted into demultiplexed compressed fastq files using bcl2fastq v1.8.2 Conversion Software (Illumina). The reads were trimmed and quality-filtered using TrimmingReads.pl [29]. Only reads with mapping quality above 20 were retained.

### 2.3. Mapping the WGS Data to Bacterial Genomes

**Figure 1:**
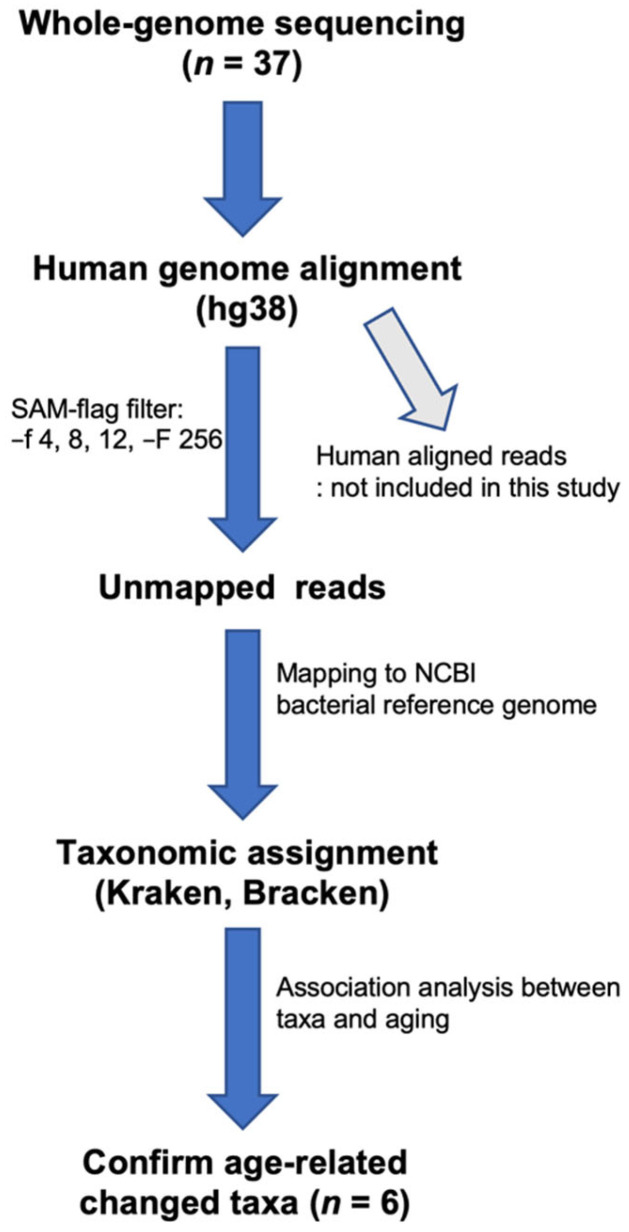
Workflow of the human DNA filtering and bacterial identification procedure. Reads that mapped to the human reference genome sequence or aligned with human sequences in the nt database were removed. The taxonomic classification of the reads was performed using Kraken and Bracken, with the help of a microbial genome database.

We separated human and non-human reads and used the latter as candidate microbial reads for taxonomic profiling of microbial communities. To identify potential microbial reads, this analysis was based on PathSeq protocols [30], with a few modifications. First, the sequencing reads were aligned to the human reference genome (version hg38; http://genome.ucsc.edu/, accessed on 2 January 2017). To exclude potential human reads, the reads were mapped to the NCBI assembled human reference (hs_ref_GRCh38.p7, hs_alt_CHM1_1.1, https://ftp.ncbi.nlm.nih.gov/genomes/archive/old_refseq/H_sapiens/ARCHIVE/ANNOTATION_RELEASE.108/Assembled_chromosomes/seq/, accessed on 2 January 2017) and Ensembl human reference (download release87 in http://asia.ensembl.org/info/docs/tools/vep/script/vep_download.html#toolsversions, accessed on 2 January 2017) genomes using Burrows–Wheeler aligner-mem (BWA-mem; version 0.7.16), with default settings, in a paired-end manner. The remaining unmapped reads were mapped to the NCBI full set of microbial reference genomes using Kraken and Bracken, to determine the taxonomic composition of the microbial communities in the blood (Figure 1). Reads that aligned with humans were removed from further analyses. Taxonomic classification of unmapped reads was performed using Kraken (version 1.0, http://ccb.jhu.edu/software/kraken, accessed on 10 November 2017) [31], Bayesian Reestimation of Abundance after Classification with KrakEN (Bracken; version 1.0; http://ccb.jhu.edu/software/bracken/, accessed on 10 November 2017) [32] and a custom-built database constructed from all bacterial genomes deposited in the NCBI RefSeq database (https://ftp.ncbi.nlm.nih.gov/genomes/archive/old_refseq/Bacteria, accessed on 7 January 2017), with default parameters and no k-mer removal. The number of reads classified for each taxon was recorded as the mapping counts and relative taxa abundance values. We found an abundance level of 1155 species, belonging to 488 genera, 207 families, 42 classes, and 23 phyla, in our data.

### 2.4. Genomic Coverage

To calculate the genomic coverage of the bacterial mapped reads, we used source codes from the WGSpipeline (http://github.com/zhangch/WGSpipeline/Scripts/Profiling.sh,~/calVar.R (accessed on 25 November 2017)) [30]. Briefly, bowtie2 [33] was used as the aligner to map the reads to each bacterial genome, and positive bacterial identification was determined by applying the options and parameters used in the source code.

### 2.5. Taxonomic Analysis

To find associations between aging and microbial community abundance, generalized linear models implemented in the multivariate association with linear models (MaAsLin) package [34] from RStudio (v.1.0.153) were used. MaAsLin is a multivariate statistical framework that identifies associations between clinical metadata and microbial community abundance. In the present study, MaAsLin was used to detect associations between microbiota composition and three age groups (Group 1: age ≤ 40 years, Group 2: age 40 to 60 years, Group 3: age > 60 years). Mixed-effect linear models using an arcsin square root transform on relative abundances were used to determine the significance of the associations between the three age groups and the microbiota. Sex and BMI were used as fixed effects, while family ID was used as a random effect in the linear regression model. The default MaAsLin parameters were used. The resulting *p*-values were corrected for multiple comparisons at each phylogenetic level and among the three age groups, using the Benjamini–Hochberg correction (FDR). FDR *q* < 0.05 or Bonferroni *p.adj* < 0.05 was considered statistically significant.

### 2.6. Statistical Analysis

Basic statistical analyses were performed using RStudio (v.1.0.153). To determine the correlation between the bacterial mapping ratio and age, sex, and family information, we applied the Spearman’s correlation test for continuous variables, Wilcoxon test for two groups, and Kruskal-Wallis for four groups, respectively, using RStudio (v.1.0.153). A plot of correlation was constructed using the stat_smooth R package. For principal component analysis (PCA), we adopted a compositional data (CoDa) analysis approach (http://github.com/ggloor, accessed on 25 November 2017) for count zero multiplicative (CZM) normalization of count data. Zero count taxa at the class level were replaced with an imputed value from the zCompostions R package, using the CZM method [35]. The centered log ratio (clr) transformation was applied to the zero-replaced data set; the data were subsequently used as input for a singular value decomposition [36] while the PCA plot was drawn using the factoextra R package. Alpha and beta diversities were calculated using the QIIME2 package (version 2017.11, https://qiime2.org, accessed on 10 November 2017) [37]. Microbial richness, which measures the number of distinct taxa in each sample, was examined by calculating the Chao1 index, which is the predicted number of taxa in a sample, by extrapolating the number of rare organisms that may have been missed due to under-sampling. Shannon’s index, which measures diversity by accounting for evenness, was also calculated. Bray–Curtis dissimilarity was used as a non-phylogenetic method to determine the dissimilarity between community membership and structure. The Bray–Curtis dissimilarity is robust to the presence of zeroes in a count table, as is often the case for microbiome data. Tests of significance were performed using a non-parametric *t*-test, with 10,000 Monte Carlo permutations.

## 3. Results

### 3.1. Demographics of Subjects

This study included 37 participants (18 men and 19 women) (Table 1). The mean age of the participants was 49.35 years (standard deviation = 20.30). We categorized the subjects into 3 groups according to age: young (≤40 years), middle (40–60 years), and elderly (>60 years). The mean height and BMI were significantly different among the three groups (*p* < 0.05). Other variables, including sex, weight, fasting glucose, proportion of type-2 diabetes and hypertension, showed no significant differences among the 3 groups (Table 1).

### 3.2. Identification of the Blood Microbiota Using WGS Data

**Figure 2:**
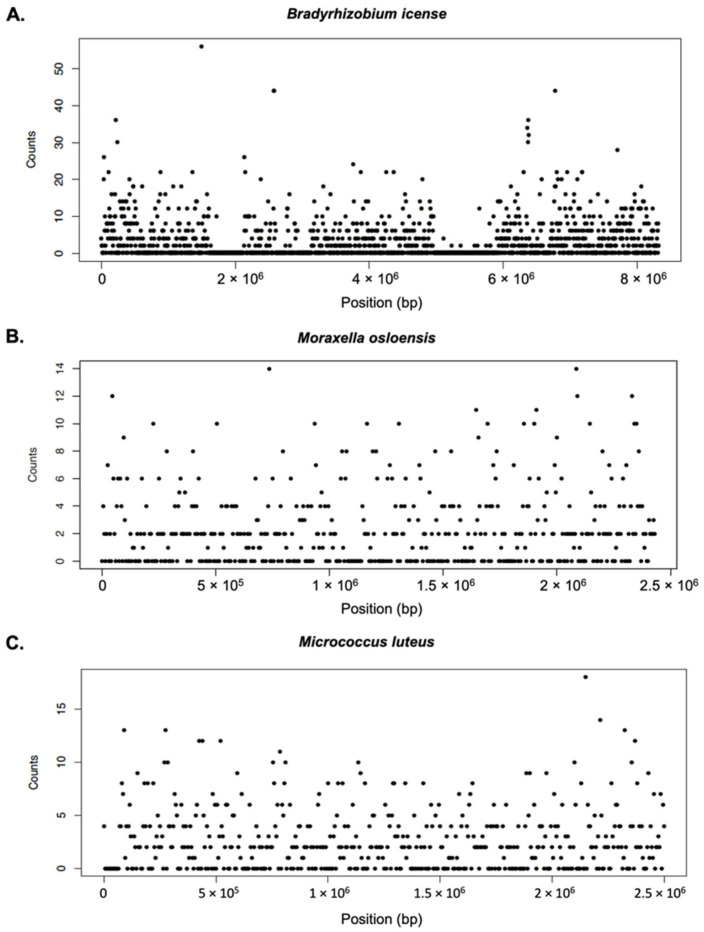
Genomic sequencing coverage. (**A**) *Bradyrhizobium* *icense*, (**B**) *Moraxella osloenosis*, and (**C**) *Micrococcus luteus*. Each window is shown by a dot and each dot represents sequence reads of 100 bp size.

To study the composition of the microbial communities, we determined the metagenome present in the blood of the family members. Profiling of the microbiota in human blood samples is limited because the blood contains mostly human DNA and a low abundance of microbial DNA. To overcome this limitation, we performed WGS on 37 blood samples, at a read depth of 30× for 10 samples, 60× for 17 samples, and 90× for 10 samples. On an average, each library produced 800 million reads, of which 97% were mapped to the standard human reference genome (Table S1). We compared the dependency of the bacterial mapping ratio on the basic characteristics of the subjects (Figure S1). There was no significant difference in sex and family relationships according to the bacterial mapping ratio affected by genomic read depth. Linear regression analysis of the bacterial mapping ratio revealed significant differences according to age (*p* < 0.05). To reduce the false detection of bacteria, according to the recommended protocol of Chao et al. [30], we evaluated the number of reads and coverage uniformity that mapped to the bacterial genomes. The data for *Bradyrhizobium* *icense*, *Moraxella osloensis*, and *Micrococcus luteus* were evenly distributed throughout the genome, providing support for positive identification (Figure 2). *Bradyrhizobium* genus is the most abundant metagenome, showing that the reads in this experiment are evenly distributed throughout the reference genome. Nevertheless, the overall read depth is low to assemble and analyze these reads to form a contig. To represent the situation with low abundancy, *Moraxella osloensis* belonging to Gammaproteobacteria and *Micrococcus luteus* belonging to Actinomycetota were randomly selected. Even in low abundance taxa, the results of mapping to their reference genome also distribute each read homogeneously, suggesting that it reflects each bacterium well.

### 3.3. Overview of the Blood Microbiota Composition

**Figure 3:**
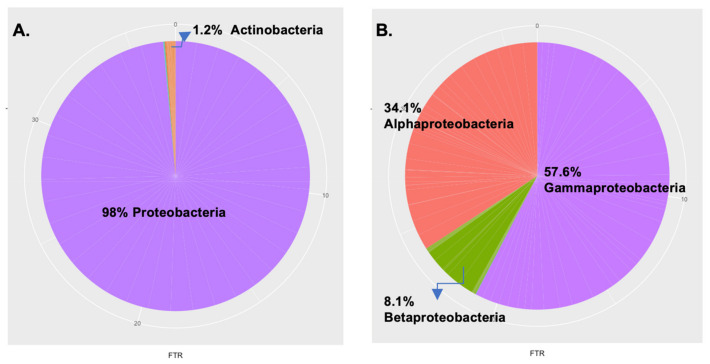
Proportion of the specific blood microbiota. The pie charts given here represent the relative abundance of the (**A**) phylum and (**B**) class level distribution of the most dominant phylum of proteobacteria in all the participants.

To assess the assembly and diversity of the blood microbiota, we performed taxonomic assignment of the bacterial sequences present in the blood of the five families. In total, we observed 23 distinct phyla, with an average of 13 ± 2 phyla per individual. Sequences of the overall population primarily belonged to the Proteobacteria (98%) and Actinobacteria (1.3%) phyla, and, to a lesser extent, to the Firmicutes and Bacteroidetes phyla (Figure 3A). The classes in the phyla Proteobacteria primarily belonged to Gammaproteobacteria and Alphaproteobacteria, and, to a lesser degree, to Betaproteobacteria and Deltaproteobacteria (Figure 3B).

**Figure 4:**
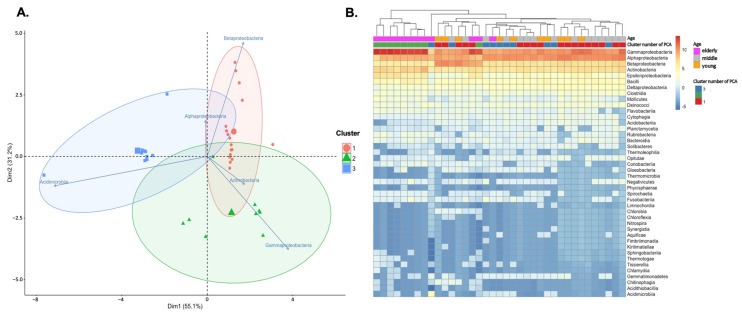
Relative abundance of class level taxa detected in the blood microbiota. (**A**) Principal component analysis (PCA) allowed for a refinement of the overall subjects into clusters 1, 2, and 3. The color indicates the identity of each sample. A PCA plot of samples correlated with the five most common classes after clr transformation (frequency > 0.01 in any sample). (**B**) Heatmap showing relative abundance of clr-transformed taxa at the class level, using the Euclidian method (dendrogram). The young were under 40 years old, middle-aged were from 40 to 60 years old, and elderly were over 60 years old; the cluster number of PCA (cluster) for each subject is indicated by the top bars.

To assess the relatedness between the microbiota and subjects, PCA and unsupervised hierarchical clustering were performed. PCA analysis using the 5 most common taxa centered on log-transformed data revealed several interesting patterns in the microbiota composition from the 3 cluster groups along PC1, which explained for 51.5% of the variance (Figure 4A). First, the classes Acidimicrobia, Alphaproteobacteria, and Betaproteobacteria were relatively enriched in clusters 1 and 3, in 17 out of the 37 samples. In contrast, Gammaproteobacteria and Actinobacteria were enriched in Cluster 2, in 10 out of 12 samples over 60 years of age. However, no effect of gender and familial relationships was observed. In addition, in unsupervised hierarchical clustering analysis, eight of the 10 eldest subjects in cluster 2 formed a distinct branch of the dendrogram with the elderly group (Figure 4B). Testing of the Bray–Curtis distances using PERMANOVA detected a significant main effect from the aged groups (*p* < 0.0001), indicating that the microbial communities detected in each age group were significantly different in composition. Therefore, we observed a difference in the composition of the microbiota according to age, rather than family unit, and the samples were divided into three age groups (young: age ≤ 40 years; middle: age 40 to 60 years; elderly: age > 60 years).

### 3.4. Blood Microbial Diversity

**Figure 5:**
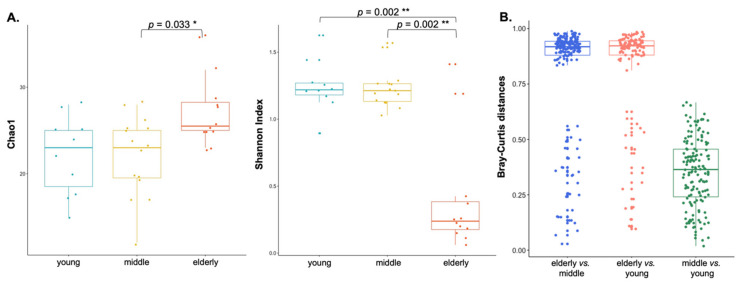
Comparisons of alpha and beta diversities in all three age groups. (**A**) Alpha diversity was measured in terms of Chao1 and Shannon indexes among the three age groups, as a qualitative trait (Dunn test, Bonferroni adjusted *p*-value). (**B**) Bray–Curtis distances show significant differences in the elderly vs. middle/young (two-tailed Student’s *t*-test using 10,000 Monte Carlo permutations, computed using QIMME’s script make_distance_boxplots.py). The central line shown in each box plot indicates the median of the data and the whiskers extend to cover the whole range of values. * *p* < 0.05 and ** *p* < 0.01.

To evaluate the potential differences in the microbial profiles of individuals according to age, we explored the richness and composition of microbial communities across the groups. Alpha diversity measures incorporating evenness of microbial taxa showed that the eldest group (elderly) had a much lower microbial taxa diversity than the other groups (Shannon index *p* = 0.002, for young vs. elderly and middle vs. elderly, Dunn test), with slightly higher richness than that in the others (Chao1 index; *p* = 0.033, for the middle vs. elderly, Dunn test), as shown in Figure 5A and Table S2. No significant differences were observed between the two groups (young and middle-aged).

To quantify beta diversity, non-polygenetic methods were used with Bray–Curtis. The mean Bray–Curtis within the elderly group was significantly higher than the between-group distances (non-parametric *p* < 0.001, two-sample *t*-test via 100,000 Monte Carlo permutations: Figure 5B). This indicates a lower diversity and a closer similarity within the elderly group paired samples (versus that between-groups). Non-parametric analysis of similarities tests (ANOSIM) demonstrated a significant difference in the overall bacterial community composition in the blood microbiota, between the elderly group and the 2 other groups (ANOSIM: Bray–Curtis, *p* < 0.001; PERMANOVA: Bray–Curtis, *p* < 0.001).

### 3.5. Association between Blood Microbial Composition and Age

**Figure 6:**
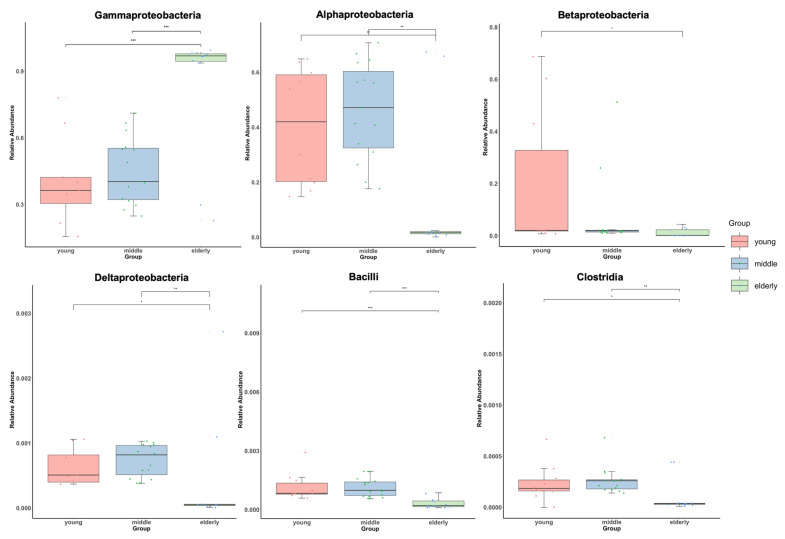
Boxplots showing a significant correlation between the three age groups and six classes compositions at the class level. The *y*-axis indicates relative abundances that were arcsine square root-transformed for taxon, with the analyses adjusted for sex and BMI. The *x*-axis indicates the three age groups; the young group had 10 individuals, the middle group had 15 individuals, and the elderly group had 12 individuals. The central line shown in each notched box plot indicates the median of the data. The detailed statistics are shown in Table S3. * *q* < 0.05, ** *q* < 0.01, and *** *q* < 0.001.

An individual’s microbiota can be classified according to the degree of aging. To identify specific microbial taxa significantly associated with age-related changes with adjustments for sex and BMI, we performed an MaAsLin analysis using a linear mixed effect model. Groups were compared pairwise, using categorical variables for age range (Figure 6). MaAsLin analysis identified 6 candidate taxa that correlated with age groups, when a FDR of *q* < 0.05 was applied (Table S3), the threshold employed in previous microbiome studies, which allows for compensation of multiple microbial taxa and comparison adjustments [34]. Among these, 6 taxa exhibited significant correlations with age (*q* < 0.05). Specifically, Gammaproteobacteria were more abundant in the elderly group (young vs. elderly: beta-coefficient = 0.61, *q* < 0.001; middle vs. elderly: beta-coefficient = 0.58, *q* < 0.001). The elderly group showed a negative correlation with the arcsine square root-transformed relative abundance of Alphaproteobacteria (young vs. elderly: beta-coefficient = −0.42, *q* = 0.012; middle vs. elderly: beta-coefficient = −0.51, *q* < 0.001) and Deltaproteobacteria (beta-coefficient = −0.01, *q* = 0.04; middle vs. elderly: beta-coefficient = −0.02, *q* = 0.002) belonging to the phyla Proteobacteria, Clostridia belonging to the phylum Firmicutes, and Bacilli belonging to the phylum Bacillota.

## 4. Discussion

In this study, we investigated unmapped reads from WGS using whole blood, to analyze the composition of the blood microbiota. To detect bacterial genomes from the WGS data, filtering processes were required to remove all possible reads originating from human DNA; this is because bacterial DNA is present at much lower fractions than human DNA in the blood. By filtering human reads from the unmapped reads, we considered only high-quality non-human reads and assigned taxonomic labels to bacterial DNA sequences using a highly accurate taxonomic sequence classification approach, based on a custom database comprising complete and high-quality reference bacterial genomes. As a result, we identified that Proteobacteria and Actinobacteria were the most abundant phyla, comprising more than 95% of the phyla in blood, which is in line with the composition results of previous studies, which were obtained using 16S rRNA sequencing [8,13]. Furthermore, because of the low coverage of bacterial genomes and sequence similarities, bacteria are often identified based on coverage of a narrow region of their genomes. Zhang et al. reported that correctly identified bacteria did not dramatically reduce the number of mapped reads with filtering steps, and the coverage across each genome was more uniform [30]. In our study, the observed microbiota coverage across each genome was uniform in many subjects. Therefore, the results obtained from our analysis were sufficiently reliable to be used for subsequent analyses.

The host genetic environment plays a role in determining microbiome variation among people [38,39]. Particularly in family studies, data on genetic relatedness among family members, gender, and age are relatively more defined than that of a random population. However, Daphna et al. reported that diet and lifestyle are more important factors in the formation of microbiome composition than human genetic characteristics [40]. In addition, sex has been flagged as a variable that significantly influences the microbiome [41,42]. In this study, among the factors reported to affect microbial composition, we found that age influenced the blood microbial fraction and composition, whereas gender and familial aggregation did not (Figure S1 and Figure 4A). PCA clustering analysis and grouping according to age range showed closer similarity in microbiota abundance in an unsupervised heatmap of all taxa at the class level. Similarly to our results, recent studies on gut and respiratory microbiomes have reported clustering patterns based on various age ranges [43,44]. In addition, the diversity of the blood microbiota was strongly associated with age in this study. A decrease in diversity in several studies, including ours, implies a general trend in the aging process [45] as well as highlights diversity as a major factor for promoting the stability of organ systems. Consequently, a decrease in diversity could reduce the protective effects of the microbiome and render a person vulnerable to infection. The elderly have a different gut or respiratory microbiome profile compared to healthy middle-aged persons, and this difference could be attributed to several factors associated with senescence, such as changes in lifestyle and dietary schedule, reduced mobility, weakened immune strength, reduced intestinal and overall functionality, altered gut morphology and physiology, recurrent infections, and the use of medications [46,47]. Changes in microbiota diversity in the blood may differ from that in other tissues, such as the gut and respiratory tract. Specifically, the intestine, skin, mouth, vagina, and respiratory tract may be vulnerable to changes, due to exposure to the external environment. Blood, on the other hand, is rarely exposed to changes in the external environment, but is always exposed to immune molecules and cells. In this respect, it is noteworthy that the blood microbiota changed depending on age in this study.

Aging is associated with a progressive decline in physiological functions, including immune system dysfunction, which results in increased susceptibility to various infections [48,49]. Many hallmarks of aging, including increased inflammatory signaling and oxidative stress, may be partly due to the dysbiotic microbiome [50,51]. Proteobacteria are gram-negative bacteria with six classes of phyla that are known to be involved in inflammation. They vary widely in morphology, function, and pathogenicity among genus species, but most of them are facultatively or obligately anaerobic in many cases. An increase in the abundance of Proteobacteria has been reported to correlate with inflammation in the elderly [52]. In our study, Gammaproteobacteria, which play a very important role in the migration and retention of antibiotic-resistant bacteria, were more prevalent in the elderly group than in the other groups. van Tongeren et al. reported that there was a correlation between increased gram-negative Gammaproteobacteria and frailty in the elderly [53]. In the gastrointestinal tract, the connection between Gammaproteobacteria and inflammation has been studied [54]. Toll-like receptor (TLR) recognizes LPS [55,56], a constituent of the cell wall of gram-negative bacteria. In addition, Ghosh et al. reported that increased TLR4 expression and circulating plasma LPS concentrations, known as metabolic endotoxemia, are associated with human aging [57]. We suggest that LPS of enriched Gammaproteobacteria could induce TLR4 expression in older individuals, because aging is considered a state of low-grade inflammation. Gram-negative LPS-negative Alphaproteobacteria are often associated with autoimmunity [58,59], although the underlying pathological mechanisms are not understood to date. *Sphingomonodaceae*, belonging to the class Alphaproteobacteria, contain glycosphingolipids (GSLs) instead of LPS [60]. Natural killer T cells specifically recognize GSLs of *Sphingomonodaceae* cell walls and dominate the innate immune response in the absence of TLR4 activation by LPS [61,62]. Little research exists regarding non-diseased populations or the increase in Gammaproteobacteria and decrease in Alphaproteobacteria in the elderly population. The opposite directions of the association with age in these two taxa and the functional implications of these differences need to be confirmed through further studies. Another microbiota, gram-positive Clostridia, belonging to the phylum Firmicutes, accounts for a substantial part of the total bacteria in the gut microbiota [63,64]. Several researchers have reported that Clostridia is a crucial factor modulating physiological, metabolic, and immune processes in the gut that appear to be necessary for maintaining normal gut immune homeostasis [65,66]. Clostridia and Deltaproteobacteria are representative taxa in the gut microbiota of Japanese adult and elderly clusters [46]. These particular microbial communities in the elderly groups, as seen in the results of this study, indicate that a healthy microbial community may be affected by age-related physiological and immunological changes.

Our study has several limitations. First, we only analyzed bacteria at the phylum and class levels. For analysis at the genus or species level, the read number of the unmapped human genome reference must be approximately 10 Gb or higher, to perform analysis such as a reference- or assembly-based analysis. As shown in Figure 2, even the species with the highest sequencing coverage are shown as discrete dots, indicating that it is difficult to assemble and analyze the species. As we reported in this manuscript, blood bacteria exist in approximately 0.1% of the host genome; therefore, it is difficult to analyze and identify species or genera with the present data. We cannot explain the rare bacteria in the blood (<1% abundance) and analyze the genera and species levels using our methods. While we generally expect that if the microbiota composition between families appears similar, it could be considered a result of living environment and diet; however, our results showed that aging plays a greater role than the significance of the sharing of living conditions and diets among families. Second, we could not explain how many dead bacteria were included in the NGS data. More research is needed to determine if the microbial DNA and RNA found in healthy human blood represent living or dead bacteria, or active or non-active bacterial taxa. Bacteriological activity in the blood can be evaluated using viability test procedures, including propidium monoazide treatment and cellular energy measurement [67]. However, there is currently no particular and reliable method for detecting live bacteria in the human blood [12]. We found that some bacteria can be present in the blood of healthy individuals who do not have any infectious diseases, such as sepsis. Therefore, even if there are no typical symptoms of infection or fever, we should consider the potential role of the blood microbiota, because its existence in the blood could play a role in the clinical symptoms. Further studies are needed to confirm the association and causation of the blood microbiota in various diseases. In particular, only the existence of infectious diseases has been inspected prior to transfusion; however, the findings of this study show that trace amounts of blood bacteria can be transmitted through blood transfusions. Therefore, more data on blood bacteria should be accumulated in the future, and a system to check these bacteria before blood transfusion should be considered. Because bacteria do not normally pass through the blood-brain barrier, it might be assumed that there are no bacteria in the cerebral fluid, but the existence of a trace amount of bacteria in the cerebrospinal fluid should be checked, as we have found the presence of bacteria in blood. Finally, our results suggest that the microbiota in the blood changes depending on the age group of the healthy population; whether this is direct or indirect, and whether age-related changes in the microbiota cause immune aging or vice versa, needs further investigation.

## 5. Conclusions

Age is a significant factor associated with changes in blood microbiota composition. Our findings helped clarify the blood microbiota composition in a healthy population for each age group. In particular, many Proteobacteria were found to be significantly higher in the blood bacteria of the elderly, which supports the theory that LPS-endotoxins for chronic diseases increase with age. Further analysis focusing on subjects who appear to have a microbiome typical of each age group would be valuable for revealing the relationships between the blood microbiome and host immunity, including the aging process. Finally, further large-scale or longitudinal studies are needed to confirm blood bacterial alterations in chronic diseases.

## Figures and Tables

**Table 1:** Baseline characteristics in study participants.

Characteristics	Overall	Young (*N* = 10)	Middle-Aged (*N* = 15)	Elderly (*N* = 12)	*p*
Age (year) ^a^	49.35 (20.30)	23.00 (5.46)	47.67 (3.68)	73.42 (6.36)	<0.0001 ***
Sex (men, %) ^b^	48.65	50.00	40.00	58.33	0.653
Weight (kg) ^a^	62.60 (13.22)	59.81 (14.54)	65.19 (14.37)	61.69 (10.90)	0.596
Height (cm) ^a^	162.69 (9.54)	168.98 (8.92)	160.90 (8.23)	159.69 (9.80)	0.043 *
BMI (kg/m^2^) ^a^	23.57 (3.85)	20.78 (3.70)	24.95 (3.64)	24.17 (3.22)	0.019 *
Glucose (mg/dL) ^a^	95.43 (22.72)	90.30 (7.36)	101.07 (32.63)	92.67 (14.67)	0.459
Type-2 diabetes (%) ^b^	8.11	0	6.67	16.67	0.349
Hypertension (%) ^b^	29.73	30	40	16.67	0.419

Data have been expressed as ^a^ mean (standard deviation) or ^b^ percentage. Groups were categorized by age (young, age ≤40 years; middle, age 40–60 years; elderly, age >60 years). *p*-value for differences between groups were assessed by means of analysis of variance test for continuous variables and chi-square test for categorical variables. BMI, body mass index. * *p* < 0.05 and *** *p* < 0.001.

## Data Availability

The datasets in the current study are available from the corresponding author upon reasonable request and the raw sequence data can be found in a public repository, the Clinical & Omics Data Archive (CODA) at the Korea National Institute of Health (accession number R000132; https://coda.nih.go.kr/search/ngs/view.do?key=1911124233078&rsltRegistRceptId=R000132&ty=RSLTTY01, accessed on 11 April 2022).

## Supplementary Materials

The following supporting information can be downloaded at https://www.mdpi.com/article/10.3390/jpm12060939/s1, Figure S1: Correlation analysis of Bacterial mapped read count ratio (Bac_m_ratio) versus Age, sex, and family information, respectively; Table S1: Alignment statistics; Table S2: Association between alpha diversity and each age group; Table S3: MaAsLin analysis for association between age-related groups and blood microbiota composition at class level.
