# Supplementary material for: Whole-Genome Sequencing Reveals Age-Specific Changes in the Human Blood Microbiota
Source: J Pers Med. 2022 Jun 7;12(6):939. doi: 10.3390/jpm12060939 (PMC9225573; doi:10.3390/jpm12060939)
Supplement: Supplementary file 1 [file jpm-12-00939-s001.zip › jpm-1701309-supplementary.pdf]

Supplememtary Materials

Whole-genome sequencing reveals age-specific changes in the human blood microbiota

Eun-Ju Lee, Joohun Sung, Hyung-Lae Kim, Han-Na Kim

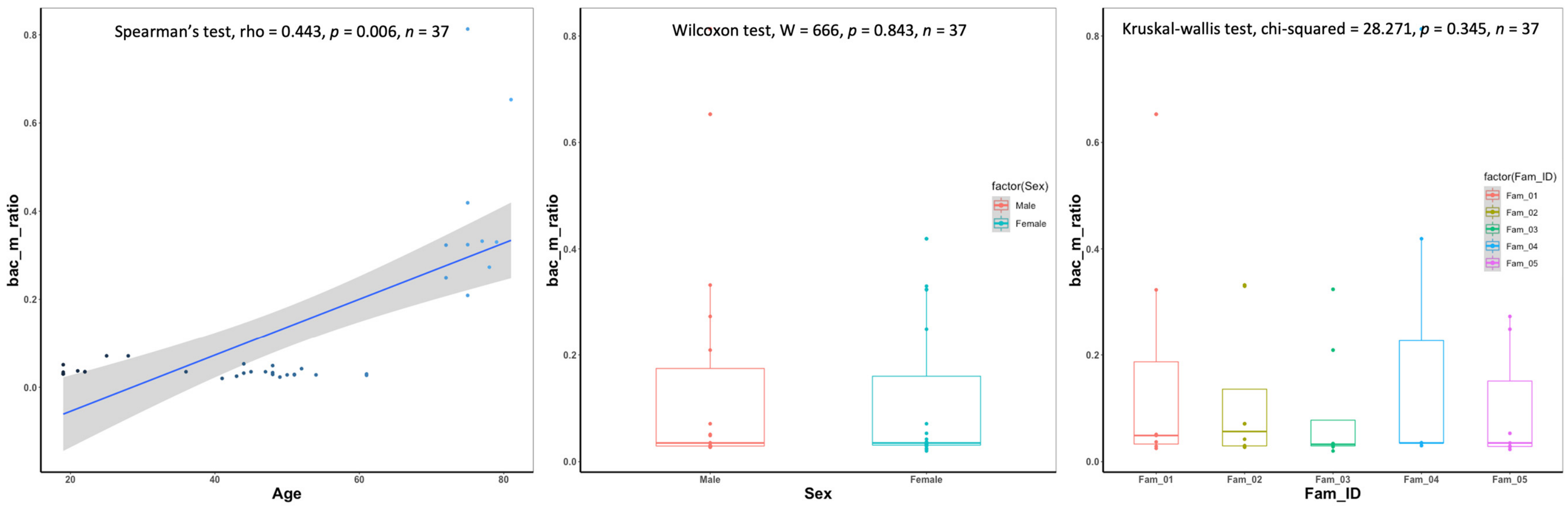

Figure S1. Correlation analysis of Bacterial mapped read count ratio (Bac_m_ratio) *versus* Age, sex, and family information, respectively. (A) Differences in bac_m_ratio by age were estimated using Spearman's correlation analysis. Blue solid line is the linear regression fitting. The shared region represents the 95% confidence interval of the predicted average Bac_m_ratio. Spearman's r = 0.443, $p = 0.006$. (B) Differences in bac_m_ratio by sex were estimated using Wilcoxon analysis. Wilcoxon's $p = 0.843$. (C) Differences in bac_m_ratio by family_ID were estimated using Kruskal-Wallis analysis. Kruskal-Wallis's chi-squared = 28.271, $p = 0.345$. The central line in each box plot indicates the median value of the data. Bacterial mapped read count ratio is calculated as ''Bac_m_ratio = (bacterial read counts/ human reference mapped read counts) × 100'.

Table S1. Alignment statistics

ID	Read depth	Mapped read count	Unmapped read count	Bacteria read count	Bacterial mapped count/mapped read count (%)	Age	Sex	Fam #	Group
T01	60×	1,798,382,502	20,935,357	541,909	0.030	61	1	F_02	elderly
T02	60×	1,740,859,059	26,157,664	461,479	0.027	61	1	F_02	elderly
T03	30×	887,366,387	13,370,628	246,933	0.028	50	1	F_02	middle
T04	60×	1,770,501,186	42,935,802	622,351	0.035	47	1	F_04	middle
T05	30×	771,775,267	20,992,644	266,267	0.035	36	1	F_04	young
T06	30×	816,493,533	22,580,121	239,128	0.029	48	1	F_01	middle
T07	60×	1,537,115,720	23,121,256	382,424	0.025	43	2	F_01	middle
T08	30×	843,725,399	22,074,593	277,499	0.033	48	2	F_03	middle
T09	60×	1,709,547,911	16,748,718	545,717	0.032	44	2	F_03	middle
T10	60×	1,588,222,534	20,437,230	321,309	0.020	41	2	F_03	middle
T11	60×	1,602,850,051	21,100,331	1,140,580	0.071	28	2	F_02	young
T12	30×	850,912,878	21,100,331	603,278	0.071	25	1	F_02	young
T13	60×	1,475,906,009	20,507,370	624,064	0.042	52	2	F_02	middle
T14	60×	1,610,789,996	22,282,703	796,126	0.049	48	1	F_01	middle
T15	30×	912,475,669	22,510,947	462,449	0.051	19	1	F_01	young
T16	90×	2,376,362,017	29,905,874	19,329,647	0.813	75	1	F_04	elderly
T17	90×	2,439,281,636	104,229,691	10,218,573	0.419	75	2	F_04	elderly
T18	90×	2,422,096,141	77,869,725	15,824,579	0.653	81	1	F_01	elderly
T19	90×	2,367,730,960	104,425,770	7,645,610	0.323	72	2	F_01	Elderly
T20	90×	2,451,819,399	78,476,199	7,955,482	0.324	75	2	F_03	elderly
T21	90×	2,476,406,110	97,361,593	8,226,061	0.332	77	1	F_02	elderly
T22	90×	2,458,060,510	98,054,785	8,101,907	0.330	79	2	F_02	elderly
T23	90×	2,438,120,791	94,567,085	6,644,230	0.273	78	1	F_05	elderly
T24	90×	2,433,897,574	116,432,516	6,064,763	0.249	72	2	F_05	elderly
T25	90×	2,407,008,071	88,399,448	5,030,374	0.209	75	1	F_03	elderly
T26	60×	1,586,969,704	19,837,025	582,253	0.037	21	2	F_01	young
T27	60×	1,636,634,093	24,290,197	369,801	0.023	49	2	F_05	middle
T28	30×	784,321,716	17,783,127	227,739	0.029	51	2	F_05	middle
T29	30×	840,997,604	20,301,201	443,392	0.053	44	2	F_05	middle
T30	60×	1,664,062,872	19,997,641	469,795	0.028	54	1	F_05	middle
T31	60×	1,762,000,018	33,452,785	610,197	0.035	22	2	F_05	young

T32	60×	1,672,488,042	25,353,065	464,840	0.028	51	1	F_03	middle
T33	60×	1,896,772,863	41,261,065	573,353	0.030	19	2	F_03	young
T34	60×	1,784,000,480	34,476,594	620,696	0.035	45	2	F_04	middle
T35	60×	1,396,662,541	20,546,332	487,191	0.035	22	1	F_04	young
T36	30×	864,983,274	11,759,876	261,956	0.030	19	1	F_04	young
T37	30×	852,925,845	12,916,295	288,540	0.034	19	2	F_03	young

Table S2. Association between alpha diversity and each age group

Alpha diversity	K.W test	Post hoc test			
		Group comparison	Z	*P. unadj*	*P. adj* *
Shannon's index	H = 15.249 (*P* < 0.001)	young vs middle	0.302	0.763	1.0
		young vs elderly	3.366	0.001	0.002
		middle vs elderly	3.403	0.001	0.002
Chao1	H = 7.858 (*P* = 0.0197)	young vs middle	-0.011	0.99	1.0
		young vs elderly	-2.305	0.021	0.063
		middle vs elderly	-2.537	0.011	0.033

K. W. test, Kruskal-Wallis test

* Multiple comparison *p*-values adjusted with the Dunn-Bonferroni post hoc method.

young (≤40 years), middle (40–60 years), and elderly (>60 years).

Table S3. MaAsLin analysis for association between age-related groups and blood microbiota composition at class level

Feature	Group	coefficient	*p* value	*q* value
Gammaproteobacteria	middle *vs.* elderly	0.5840	0.000000	0.000004
Gammaproteobacteria	young *vs.* elderly	0.6142	0.000001	0.000039
Alphaproteobacteria	middle *vs.* elderly	-0.5058	0.000017	0.000207
Bacilli	middle *vs.* elderly	-0.0155	0.000012	0.000207
Bacilli	young *vs.* elderly	-0.0165	0.000040	0.000720
Clostridia	middle *vs.* elderly	-0.0084	0.000091	0.000815
Deltaproteobacteria	middle *vs.* elderly	-0.0153	0.000283	0.002038
Alphaproteobacteria	young *vs.* elderly	-0.4192	0.001076	0.012906
Betaproteobacteria	young *vs.* elderly	-0.3239	0.001553	0.013973
Clostridia	young *vs.* elderly	-0.0063	0.006419	0.038516
Deltaproteobacteria	young *vs.* elderly	-0.0128	0.006017	0.038516

young; age ≤ 40 years, middle; age 40 to 60 years, elderly; age > 60 years

coefficient: arcsine square root transformed regression beta was calculated using MaAsLin.

q value: FDR *q*-values were calculated using Benjamini-Hochberg correction.
